# Should CINAHL be used as one of the main databases for evidence synthesis of health services intervention?

**DOI:** 10.1002/cesm.12019

**Published:** 2023-07-12

**Authors:** Teerapon Dhippayom, Natnicha Rattanachaisit, Apinya Wateemongkollert, Rawiwan Napim, Nathorn Chaiyakunapruk

**Affiliations:** ^1^ The Research Unit of Evidence Synthesis (TRUES), Faculty of Pharmaceutical Sciences Naresuan University Phitsanulok Thailand; ^2^ Faculty of Pharmaceutical Sciences Naresuan University Phitsanulok Thailand; ^3^ Department of Pharmacy Lampang Hospital Lampang Thailand; ^4^ Naresuan University Library Naresuan University Phitsanulok Thailand; ^5^ Department of Pharmacotherapy University of Utah College of Pharmacy Salt Lake City Utah USA; ^6^ IDEAS Center, Veterans Affairs Salt Lake City Healthcare System Salt Lake City Utah USA

**Keywords:** CINAHL, database, health services intervention, search

## Abstract

**Introduction:**

CINAHL is not listed as one of the minimum databases for systematic review (SR) of interventions in the Methodological Expectations of the Cochrane Intervention Review.

**Objective:**

To determine additional studies uniquely identified from the CINAHL search in SR of health services interventions (HSI).

**Methods:**

We searched PubMed from inception to October 1, 2022 to identify SRs of HSI that determined clinical or humanistic outcomes of HSI and used CINAHL. Out of 5655 Systematic reviews identified, we randomly selected 374 SRs and extracted all primary studies included. We then explored the bibliographic databases in which the journals of those studies were indexed. The outcome of interest was the number of studies uniquely available in CINHAL. We also performed a subgroup analysis based on the type of HSI. We performed descriptive statistics to report the study outcomes using Excel (Microsoft 365).

**Results:**

A total of 7550 primary studies were identified from the 374 Systematic reviews that met the inclusion criteria. Of these studies, 7380 were journal publications that have been indexed in MEDLINE/PubMed (75.1%), Scopus (74.5%), Sciences Citation Index, SCI (54.7%), Embase (48.1%), and CINAHL (34.9%). Only 83 out of 7380 (1.1%) studies were published in journals that were uniquely indexed in CINAHL. The percentage of studies that were only available in other databases was 9.7% (Scopus), 4.3% (MEDLINE/PubMed), 1.6% (SCI), and 0.3% (Embase). The number of studies that were unique to CINAHL in specific types of HSI were: 24/1570 (1.5%) for community health services, 20/1520 (1.3%) for preventive health services, 45/3624 (1.2%) for patient care, 8/1173 (0.7%) for mental health services, and 18/2804 (0.6%) for rehabilitation.

**Conclusion:**

The gain of CINAHL to identify unique primary studies for SR of HSI appears minimal. The impact of missing studies uniquely available in CINAHL on SR summary or magnitude of effect estimates from meta‐analysis requires further investigation.

## 1 INTRODUCTION

Health service, as defined by the World Health Organization, is a service provided by healthcare professionals or others under their supervision to promote, maintain, and restore health [1]. A wide array of health services interventions (HIS) exists across diverse medical conditions, encompassing areas such as rehabilitation, community care, mental health services, and smoking cessation. Generally, the process of collecting information from the primary studies and synthesizing evidence of HSI is in accordance with the systematic review guidelines for health interventions. However, HSI differs from medication interventions in various aspects, such as the intervention's complexity, the effect of the service providers, and the published sources of the research papers. Although the current Cochrane Handbook for Systematic Reviews of Interventions has added a new section for complex interventions [2], most of the recommended approaches in various steps were based on medication intervention. Considering the fundamentals of systematic review, it is important to ensure that studies relevant to the research question are comprehensively collected to prevent bias that may arise from analyzing results of incomplete data [3]. A good search requires an appropriate search strategy and a broad range of data sources. The Methodological Expectations of the Cochrane Intervention Review (MECIR) explicitly stated the necessity to search the following three main databases: Cochrane Central Register of Controlled Trials (CENTRAL), Medical Literature Analysis and Retrieval System Online (MEDLINE), and Excerpta Medica Database (Embase) [4]. Other databases such as Cumulative Index to Nursing & Allied Health Literature (CINAHL), Allied and Complementary Medicine (AMED), and PsycINFO are classified as subject‐specific databases. CINAHL is a database for nursing and multidisciplinary health care, AMED for alternative treatments, and PsycINFO for psychiatric and psychiatric studies [5].

The CINAHL database is a bibliographic database hosted by EBSCO that compiles studies published in more than 3,800 journals of nursing and allied health with study data dating back to 1961 [6]. A previous study by Wright et al revealed that CINAHL is an important database for systematic reviews of qualitative studies, with a median of 9.09% to find unique studies (ranging from 5.0% to 33.0%) [7]. Considering the extensive range of HSI covered by CINAHL, it is reasonable to assume that CINAHL contains a significant amount of unique HSI studies that are not indexed in the three primary databases recommended by MECIR.

A few previous studies have been conducted to explore the search yields of CINAHL compared with other databases for specific areas of HSI. Meseley et al. conducted a study comparing the indexing of reports for randomized controlled trials (RCTs) of physiotherapy interventions across eight bibliographic databases, including CINAHL [8]. This study included 30 Cochrane reviews with a total of 281 RCTs and found that the Physiotherapy Evidence Database (PEDro) and CENTRAL provided the most comprehensive indexing for physiotherapy RCTs. Notably, the proportion of RCTs indexed in CINAHL was lower compared to those indexed in PEDro, CENTRAL, PubMed, and EMBASE. In another study conducted by Bahaadinbeigy et al, CINAHL was compared with other databases, that is, Embase and MEDLINE [9]. The findings showed that when conducting a search for telemedicine across various medical conditions, CINAHL was able to identify 8.97% of unique telemedicine studies that were not indexed in MEDLINE and Embase. Considering the different methods used in both studies and the difference in the findings, it is still inconclusive to justify the advantage of CINAHL to identify unique HSI studies. Knowing the unique search yields of HSI studies from CINAHL could help to determine whether CINAHL should be recommended as one of the main databases for systematic reviews of HSI. Therefore, the objective of this study was to identify primary studies for systematic reviews of HSI that are uniquely indexed in CINAHL database.

## 2 METHODS

This is a cross‐sectional survey study. The population of this study was systematic reviews of HSI. We used the following search terms in PubMed: systematic review [Title/Abstract] AND health services [MeSH] AND CINAHL. We found 5655 systematic reviews that were indexed in PubMed from inception to October 1, 2022.

### 2.1 Samples

Our population is nonhuman subjects and contained no prior information regarding the population variance or proportion. We then decided to choose a precision‐based sample size calculation to determine the appropriate sample size for the known population using the following formula [10]:

$$n=\frac{N}{1+N{\left(d\right)}^{2}},$$

where *N* is the total population (5655) and *d* is the margin of error or precision (5%).

The sample size required, after applying the above formula, was 374 systematic reviews. We performed a simple random sampling of the 5655 systematic reviews by using a random number generated by Excel (Microsoft 365). NR and AW screened the random list for 374 systematic reviews that met the following inclusion criteria: (1) being a systematic review that investigated the effects of HSI; (2) using CINAHL to search for relevant studies; and (3) reporting either clinical or humanistic outcomes. Systematic reviews were excluded if they: (1) explored the effects of surgical procedures, diagnostic tests, and medical devices; (2) studied HSI as part of other interventions; or (3) did not provide reference information of the included primary studies. Disagreements regarding eligibility were discussed and resolved with TD.

### 2.2 Data collection

NR and AW conducted the extraction of the search databases utilized in the 374 systematic reviews, along with determining whether they were meta‐analyses. NR and AW classified the types of HSI of these systematic reviews based on the MeSH terms reported in PubMed for a maximum of three HSI types. Trained research assistants (RA) retrieved all primary studies of the 374 systematic reviews. The RAs then compiled a list of published journals of these studies and manually identified their indexed databases by referring to the journals or publishers' websites. A health sciences librarian, RN, was responsible for the completion and verification of the indexed databases for each journal. Subsequently, the indexed databases of the journals were connected with the original studies for data analysis.

### 2.3 Data analysis

We compiled a list of the search databases that have been used by more than 10% of the 374 systematic reviews. We used descriptive statistics, that is, mean and standard deviation (SD), to report the average number of primary studies included in the systematic reviews. We calculated the number and proportion of primary studies that were uniquely indexed in each database by filtering the existence in other databases. We also conducted subgroup analyses of the unique records of each database by different types of HSI based on the 374 systematic reviews. All analyses were performed in Excel (Microsoft 365).

## 3 RESULTS

### 3.1 Characteristics of the included systematic reviews

The included 374 systematic reviews were published between 1997 and 2022, 238 (63.6%) of which were meta‐analyses (Table 1). The most common types of systematic review of HSI, based on MeSH, among the samples of our study were patient care (51.1%), rehabilitation (37.7%), community health services (21.9%), preventive health services (19.3%), and mental health services (15.5%). All of the 374 systematic reviews searched MEDLINE/PubMed. Other search databases, excluding CINAHL, among the included systematic reviews were Embase (66.3%), CENTRAL (58.3%), PsycINFO (36.1%), Web of Science (29.7%), and Scopus (19.3%).

**Table 1:** Characteristics of included systematic review.

Characteristics	Number (%), *n* = 374
Type of systematic review	
Systematic review	136 (36.4%)
Meta‐analysis	238 (63.6%)
Type of health services intervention	
Patient care	191 (51.1%)
Rehabilitation	141 (37.7%)
Community health services	82 (21.9%)
Preventive health services	72 (19.3%)
Mental health services	58 (15.5%)
Others	1 (0.3%)–20 (5.3%)^a^
Searching database	
MEDLINE/PubMed	374 (100.0%)
Embase	248 (66.3%)
CENTRAL	218 (58.3%)
CINAHL	374 (100.0%)
PsycINFO	135 (36.1%)
Web of Science	111 (29.7%)
Scopus	72 (19.3%)
Others	3 (0.8%)–30 (8.0%)^b^

Abbreviations: HSI, health services interventions; SR, systematic review.

^a^ Other HSI types ranged from health services for transgender (1 SR) to child care (20 SR).

^b^ Other searching databases ranged from CAB abstract (3 SR) to PEDro (30 SR).

### 3.2 Studies unique to databases

**Figure 1:**
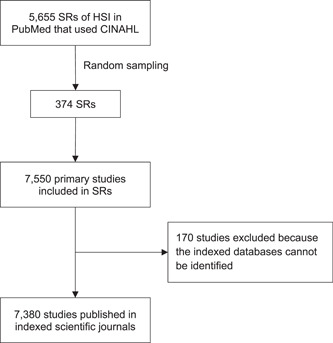
Study selection flow diagram. HSI, health services interventions; SR, systematic review.

A total of 7380 primary studies were included in the 374 systematic reviews (Figure 1), with a mean (SD) of 20.2 (18.2) studies. These studies were published in 1918 journals. Of the seven databases that were searched by the 374 systematic reviews, only five were selected to determine the indexed studies. CENTRAL was excluded due to its limited scope as a repository because it does not encompass comprehensive bibliographic information on published works; whereas PsycINFO was removed because it was a subject‐specific database. Web of Science is a collection of databases and has been replaced with the Science Citation Index (SCI). The 7380 studies were indexed in MEDLINE/PubMed (75.1%), Scopus (74.5%), SCI (54.7%), Embase (48.1%), and CINAHL (34.9%). For the unique studies in each database, 83 (1.1%) were exclusively available in CINAHL (Table 2). Out of the five databases, Scopus contains the highest number of 719 (9.7%) unique studies that were included in our sample of systematic reviews of HSI.

**Table 2:** Number of primary studies unique to each database.

Primary studies of HSI SR	Unique to CINAHL	Unique to MEDLINE/PubMed	Unique to Embase	Unique to Scopus	Unique to SCI
Total (*n* = 7380)	83 (1.1%)	319 (4.3%)	24 (0.3%)	719 (9.7%)	115 (1.6%)
Type of health services					
Patient care (*n* = 3624)	45 (1.2%)	173 (4.8%)	10 (0.3%)	367 (10.1%)	39 (1.1%)
Rehabilitation (*n* = 2804)	18 (0.6%)	95 (3.4%)	11 (0.4%)	296 (10.6%)	30 (1.1%)
Community health services (*n* = 1570)	24 (1.5%)	83 (5.3%)	4 (0.3%)	160 (10.2%)	26 (1.7%)
Preventive health services (*n* = 1520)	20 (1.3%)	70 (4.6%)	6 (0.4%)	132 (8.7%)	20 (1.3%)
Mental health services (*n* = 1173)	8 (0.7%)	39 (3.3%)	2 (0.2%)	136 (11.6%)	14 (1.2%)

Abbreviations: HSI, health services interventions; SR, systematic review.

### 3.3 Subgroup analysis: Types of health services

Considering the number of unique studies in different types of HSI, twice the proportion of unique studies in CINAHL can be identified in systematic reviews of community health services (1.5%) compared to mental health services (0.7%) and rehabilitation (0.6%). The variation in the proportion of unique primary studies across different types of HSI seems less pronounced in the other databases (Table 2).

## 4 DISCUSSION

Health services encompass a wide range of health interventions. Systematic reviews of HSI should, therefore, cover the variation of this area to ensure that the potentially relevant studies are comprehensively collected to provide the most valid evidence. In this study, we have overcome the limitations of previous studies that focused only on specific fields of HSI by broadening the scope of the study to cover all HSI fields. The strength of our study is that we have included a substantial number of systematic reviews of HSI. According to the sample size, sampling technique, and variation of the included systematic reviews, we are certain that our samples are adequately represented systematic reviews of HSI. We hypothesized that the number of primary studies uniquely available in CINAHL would be substantial to support its use as one of the main databases for systematic reviews of HSI. Unfortunately, a relatively low number of unique studies in CINHAL appears not to align with our assumption.

However, we have discovered some interesting findings that are worth remarking on here: (1) the number of unique studies in Embase, which is one of the recommended main databases for health intervention, was the lowest among the five databases; and (2) Scopus provided the highest number of unique studies compared with other databases despite less than 20% of the included systematic reviews used this database to search for primary studies. It is not surprising to see a large number of unique studies in Scopus because it is one of the biggest databases that covers 23,452 peer‐reviewed journals across a wide variety of disciplines [11]. Although the publisher stated that Embase contains 2900 unique journals [12], of our studies suggested that not many of these journals published studies in HSI areas.

Despite using similar methods to ours, the previous study conducted by Meseley et al had a specific focus on physiotherapy interventions [8]. The most comprehensive database identified in this study, that is, PEDro, is also a subject‐specific database. Therefore, it would be inappropriate to directly compare the findings of our study with those of Meseley et al. While the study conducted by Bahaadinbeigy et al was able to identify unique studies in CINAHL, Embase, and MEDLINE [9], it did not investigate the relevance of these studies to the field nor impose restrictions on the study designs. Consequently, the reported studies from each database may not be eligible for a systematic review of telemedicine. This could potentially account for the slightly higher proportion of unique studies found in CINAHL (8.97%) compared to our study.

Having a large number of included primary studies in the samples of systematic reviews allows us to conduct a subgroup analysis based on the types of HSI to explore the potential of gaining unique studies from CINAHL in certain areas of HSI. It is evident that CINAHL exhibited a distinct advantage in identifying unique studies in the broader field of HSI such as community health services, preventive health services, and patient care than in rehabilitation and mental health services. This is probably because the scope of nursing and allied health journals indexed in CINAHL is more general than topic‐specific to certain types of HSI.

### 4.1 Implication into practice

According to the findings from our study, unique studies always existed in every database, and it is almost impossible to search all available general databases to obtain studies that could not be identified in others. For the comprehensive collection of relevant primary studies, we recommend that systematic reviewers consider searching as many bibliographic databases as possible. Numerous search records could be screened using artificial intelligence‐aiding software [13], and duplicates can be efficiently removed if different databases are searched under the same platforms/publishers [14]. We recommend that researchers prioritize selecting databases based on their capacity to provide unique studies for systematic reviews of HSI, as indicated by the findings of our study. It is also important to use techniques other than database searching as recommended in the latest PRISMA 2020 statement [15] to ensure that unique studies from missing databases, as well as grey literature, are well captured.

### 4.2 Limitations

Our study has a limitation concerning the comprehensiveness of the search strategy, which may not encompass the most recent systematic review of HSI. This limitation arises from our reliance on MeSH terms for health services, which introduces a time lag as relevant studies need to be indexed within the MeSH database. We opted not to employ free text searches due to the vast number of terms associated with HSI, making it impractical to include all relevant terms. However, we are confident that the utilization of MeSH terms for health services adequately covers the majority of HSI studies. Findings in our study were derived from systematic reviews that used CINAHL to search for relevant studies. It is likely that the authors of these systematic reviews have already decided that primary studies relevant to their research topics could be identified from CINAHL. This infers confounding by indication, which might lead to an overestimation of the number of unique studies in CINHAL shown in our study. However, this limitation would have little impact on the implication of our findings since the unique studies identified in CINAHL are already relatively low. We did not include all systematic reviews irrespective of the use of CINAHL searching because it would be impractical to perform a search in CINAHL using the same search strategy and then screen for studies that meet the inclusion criteria of each SR to identify studies that are unique to CINAHL.

## 5 CONCLUSIONS

The added value of using CINAHL to identify distinct primary studies for synthesizing evidence on Health Service Interventions (HSI) seems limited, which undermines our initial assumption of recommending CINAHL as a primary search database for systematic reviews on HSI. However, this doesn't mean that CINAHL should be omitted. Instead, the findings suggest that unique studies identified from CINAHL in general HSI and certain types of HSI were higher than other databases. We, therefore, still recommend that CINAHL should still be considered when searching for systematic reviews of HSI. To examine the potential benefits of CINAHL, we encourage that further studies should be conducted to investigate the impact of missing studies uniquely available in CINAHL on the summary of systematic reviews or the magnitude of effect estimates from meta‐analyses of HSI.

## AUTHOR CONTRIBUTIONS

**Teerapon Dhippayom**: Conceptualization; data curation; formal analysis; funding acquisition; investigation; methodology; project administration; writing—original draft; writing—review and editing. **Natnicha Rattanachaisit**: Data curation; methodology; writing—review and editing. **Apinya Wateemongkollert**: Data curation; methodology; writing—review and editing. **Rawiwan Napim**: Resources; validation; writing—review and editing. **Nathorn Chaiyakunapruk**: Supervision; writing—review and editing.

## CONFLICT OF INTEREST STATEMENT

The authors declare no conflicts of interest.

## PEER REVIEW

The peer review history for this article is available at https://www.webofscience.com/api/gateway/wos/peer-review/10.1002/cesm.12019.

## Data Availability

Data available on request from the authors.
